# Outpatient influenza antivirals in a distributed data network for influenza surveillance

**DOI:** 10.1111/irv.12598

**Published:** 2018-08-22

**Authors:** Noelle M. Cocoros, Genna Panucci, Nicole Haug, Carmen Maher, Marsha Reichman, Sengwee Toh

**Affiliations:** ^1^ Department of Population Medicine Harvard Pilgrim Health Care Institute Boston Massachusetts; ^2^ US Food and Drug Administration Silver Spring Maryland

**Keywords:** infectious disease surveillance, influenza, medical claims

## Abstract

Electronic data collected from routine health care can be used for public health surveillance. To examine the Sentinel System, a distributed data network of health plans, as a source for influenza surveillance, we compared trends in outpatient prescription dispensings of influenza antivirals in Sentinel to trends in CDC's ILINet and NREVSS systems over five seasons. There were 2 102 885 dispensed prescriptions of oseltamivir capsules, 494 188 of oseltamivir powder, and 7955 of zanamivir. Across all seasons, the magnitude and timing of peaks in drug utilization were highly comparable to those in ILINet and NREVSS. Oseltamivir capsules and powder were well correlated with ILINet and NREVSS. This lays the foundation for further exploration of Sentinel's utility for influenza surveillance.

## 1 BACKGROUND

In the United States, public health authorities utilize numerous data sources to monitor the annual influenza season (October through May). Because most people with influenza or other respiratory viruses that co‐circulate do not seek medical care—and those who do seek are not necessarily tested—sources ranging from syndromic surveillance to laboratory testing data are necessary to gauge the timing and severity of the season. Simonsen and colleagues have previously recommended the use of claims data to enable, among other things, local‐level estimates of influenza activity.^1^ Viboud and colleagues also demonstrated that an algorithm for influenza‐like illness (ILI) applied to medical claims data correlated very well with local and regional ILI data as well as laboratory data.^2^

The US Food and Drug Administration (FDA)'s Sentinel System is an active surveillance system that uses electronic healthcare data, primarily administrative claims from commercial insurers, from multiple sources to monitor the safety of regulated medical products.^3^ FDA has envisioned Sentinel to be a national resource for evidence generation, and for it to be leveraged for other public health, uses as well as research.^4^

We examined whether claims data for outpatient dispensings of prescription influenza antiviral drugs in the Sentinel System might serve as an additional source of influenza surveillance data. We calculated the rate of incident influenza antiviral drug prescriptions dispensed over multiple influenza seasons. We then compared trends in Sentinel to those in the Centers for Disease Control and Prevention (CDC)'s ILINet system and National Respiratory and Enteric Virus Surveillance System (NREVSS), which are part of routine national influenza surveillance. This work was conducted as a proof of concept, laying the groundwork for the potential future use of Sentinel for influenza surveillance.

## 2 METHODS

From January 2010 through December 2015, we identified outpatient prescription dispensings of oseltamivir (capsule and powder form separately) and zanamivir from 16 Sentinel Data Partners. Health plan members of all ages were included if they met the following criteria: medical and drug coverage for ≥90 days prior to the dispensing of interest and no influenza antiviral dispensing in the prior 45 days (i.e, we captured incident dispensings). We included all valid prescription dispensings per member (a member could contribute >1 treatment episode of interest if inclusion/exclusion criteria were met) and used an episode gap of 10 days, meaning dispensings <11 days apart based on days supply were treated as a single dispensing. We used National Drug Codes (NDCs) from the First Databank (San Francisco, California) to identify prescription dispensings for oseltamivir and zanamivir. We stratified the data by age and month‐year of dispensing date.

ILINet is a national network of healthcare providers maintained by states and CDC. Each week during influenza season, ~2000 outpatient healthcare providers voluntarily report on the total number of patients seen for any reason and the number of those patients with ILI. ILI is defined as fever and cough and/or sore throat without a known cause other than influenza. We downloaded data for the same time period from the CDC website by surveillance week, as defined in the Morbidity and Mortality Weekly Report (MMWR), and then manually converted the data from MMWR week to month‐year. We assigned weeks that crossed 2 months to the month where ≥4 days of the week occurred. We calculated the unweighted proportion of encounters with ILI across all age groups. CDC's NREVSS provides testing and results data from public health and clinical laboratories on several viruses including influenza. We downloaded data from January 2010 through October 2015 (public health and clinical laboratories combined) from the website by MMWR week and converted them to month‐year in the same way as ILINet data.

We plotted the prescription dispensing rate data, the proportion of ILI among encounters in ILINet, and the proportion of positive tests among those tested for influenza from NREVSS by month‐year and visually compared timing and magnitude of trends in the two systems for each season. We calculated Pearson correlation coefficients for the full study period for the three different dispensing types with respect to ILINet and NRVESS data, separately, using SAS version 9.4. This work was conducted under the Sentinel System as a public health activity, not research, and is therefore not under the purview of IRB.^5^

## 3 RESULTS

Over the study period, among 101 947 808 eligible members, there were 2 102 885 episodes of oseltamivir capsules, 494 188 of oseltamivir powder, and 7955 of zanamivir in the participating Sentinel Data Partners. As shown in Table 1, the influenza antiviral prescription dispensing data in Sentinel yielded expected results. For example, of the little zanamivir use, almost none was in children under 5 years for whom the drug is not approved. Adults were more likely to receive oseltamivir capsules, and children were more likely to receive powder. On average, there was one dispensing per user and 6‐7 days supplied per dispensing, depending on formulation.

**Table 1:** Outpatient influenza antiviral episodes in the Sentinel System, January 2010‐December 2015

	New users	New treatment episodes	Days supplied per dispensing	Dispensing per new user
Oseltamivir capsules				
All ages	1 987 276	2 102 885	5.70	1.07
<5y	44 119	44 572	6.06	1.01
5‐18y	398 317	416 167	5.68	1.05
>18y	1 553 292	1 642 146	5.69	1.06
Oseltamivir powder				
All ages	459 758	494 188	6.40	1.08
<5y	205 789	214 037	6.41	1.05
5‐18y	261 539	274 756	6.39	1.06
>18y	5353	5395	6.33	1.02
Zanamivir				
All ages	7559	7955	7.33	1.06
<5y	17	17	5.12	1.00
5‐18y	1917	1958	7.03	1.03
>18y	5653	5980	7.43	1.07

**Figure 1:**
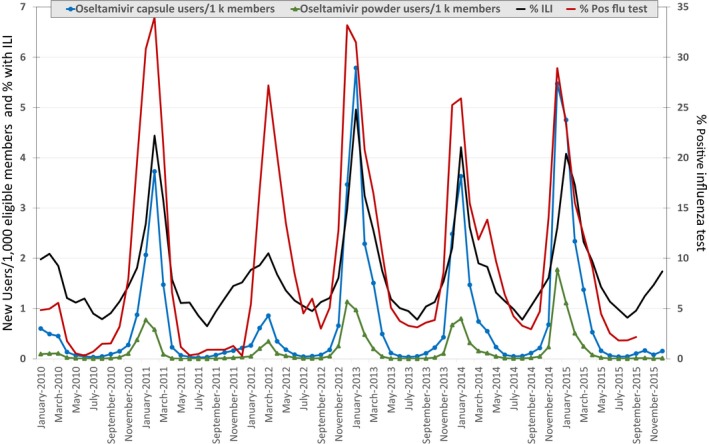
Dispensings of oseltamivir in the Sentinel System compared with the proportion of ILI encounters in ILINet and positive influenza test results in NRVESS, January 2010‐December 2015 NREVSS

When the monthly rates of outpatient oseltamivir prescription dispensings were compared with outpatient ILI trends and laboratory test results, we observed excellent overlap with respect to timing (Figure 1). Further, the general magnitude—or severity—of each season, as depicted in the ILI data, is also evident in the Sentinel dispensing data. For example, the 2011‐2012 season was mild and we correspondingly observed a lower rate of oseltamivir use compared with other seasons. We observed good correlation between the dispensing data of oseltamivir capsules and oseltamivir powder and ILI data (*ρ* = 0.89 and 0.75, *P*‐values <0.0001), and influenza testing data (*ρ* = 0.83 and 0.82, *P*‐values <0.0001). Not surprisingly given the relatively infrequent use of zanamivir, the data were less well correlated with ILI (*ρ* = 0.54, *P*‐value <0.0001) and influenza testing data (*ρ* = 0.51, *P*‐value <0.0001).

## 4 DISCUSSION

As well‐established components of the national influenza surveillance system, ILINet and NREVSS are often used as gold standards against which other data sources are compared and validated. In our analysis of five influenza seasons, we observed that the trends in prescription dispensings of influenza antivirals in the Sentinel System were highly comparable to trends in the percentage of ILI encounters among outpatient encounters as reported to ILINet and the percentage of tests positive for influenza as reported to NREVSS from public health and clinical laboratories. The oseltamivir dispensing data were also highly correlated with the ILINet and NREVSS data. Further, and not surprisingly, the descriptive data indicate we have accurately captured episodes of oseltamivir and zanamivir during influenza seasons. While this analysis was straightforward, it demonstrates that influenza antiviral dispensing data in Sentinel may be a useful source of influenza surveillance data in the future.

Two of the major strengths of the Sentinel System are its size and geographical coverage. As of mid‐2018, there are more than 14 billion pharmacy dispensings and more than 13 billion medical encounters from 2010 to 2017 across the Data Partners. Nearly 67 million active members are currently contributing data. The Data Partners serve primarily commercially insured individuals (Medicare became a part of Sentinel after this analysis was conducted). In addition, while the data are routinely updated on a quarterly basis by the largest partners, and the data have a lag of approximately 6 months, the Sentinel System has previously demonstrated the capability to obtain “fresher” data with a lag time of 6 weeks to assess influenza vaccine safety.^6^ Outpatient pharmacy data are routinely available within 2 weeks. Finally, Sentinel data have the potential to provide local‐level data on drug dispensings, vaccine utilization, and outcomes like influenza illness as member zip code is available. The ability to examine large‐scale surveillance data on a regional or local level could be a major addition to national influenza surveillance efforts, although in this feasibility analysis we looked across all available regions.

One limitation to consider when interpreting our results is that CDC tracks ILINet and NREVSS data by MMWR week. Because the Sentinel data are routinely available by month‐year, we transformed the ILINet and NREVSS data into months; we assigned weeks that crossed 2 months to the month where ≥4 days of the week occurred. We therefore could not make exact monthly or weekly comparisons between the sources in this analysis. However, the overlap in trends supports the validity of our methodology. In addition, more granular data are technically feasible in Sentinel and might be used in subsequent work.

In conclusion, we have shown that influenza antiviral dispensing data in the Sentinel System may be a new source of national influenza surveillance data. This analysis lays the groundwork for additional studies to explore and utilize Sentinel in new and important ways.

## CONFLICT OF INTEREST

The authors do not have any conflict of interests to disclose.
